# Expression of the apelin receptor, a novel potential therapeutic target, and its endogenous ligands in diverse stem cell populations in human glioblastoma

**DOI:** 10.3389/fnins.2024.1379658

**Published:** 2024-05-13

**Authors:** Thomas L. Williams, Peter Nwokoye, Rhoda E. Kuc, Kieran Smith, Anna L. Paterson, Kieren Allinson, Janet J. Maguire, Anthony P. Davenport

**Affiliations:** ^1^Experimental Medicine and Immunotherapeutics, University of Cambridge, Addenbrooke’s Hospital, Cambridge, United Kingdom; ^2^Department of Pathology, Cambridge University Hospitals NHS Foundation Trust, Cambridge, United Kingdom

**Keywords:** GPCR, apelin receptor, apelin, elabela/toddler, glioblastoma, stem cells, immunohistochemistry, neuropharmacology

## Abstract

Glioblastoma multiforme (GBM) is one of the most common and lethal forms of brain cancer, carrying a very poor prognosis (median survival of ~15 months post-diagnosis). Treatment typically involves invasive surgical resection of the tumour mass, followed by radiotherapy and adjuvant chemotherapy using the alkylating agent temozolomide, but over half of patients do not respond to this drug and considerable resistance is observed. Tumour heterogeneity is the main cause of therapeutic failure, where diverse progenitor glioblastoma stem cell (GSC) lineages in the microenvironment drive tumour recurrence and therapeutic resistance. The apelin receptor is a class A GPCR that binds two endogenous peptide ligands, apelin and ELA, and plays a role in the proliferation and survival of cancer cells. Here, we used quantitative whole slide immunofluorescent imaging of human GBM samples to characterise expression of the apelin receptor and both its ligands in the distinct GSC lineages, namely neural-progenitor-like cells (NPCs), oligodendrocyte-progenitor-like cells (OPCs), and mesenchymal-like cells (MES), as well as reactive astrocytic cells. The data confirm the presence of the apelin receptor as a tractable drug target that is common across the key cell populations driving tumour growth and maintenance, offering a potential novel therapeutic approach for patients with GBM.

## 1 Introduction

Glioblastoma multiforme (GBM) is the most common malignant primary brain tumour, representing ~45% of all incidences of glioma (Ostrom et al., 2014, 2020), with an annual prevalence of ~5 people per 100,000 of the population (Omuro and DeAngelis, 2013). GBM is highly aggressive and carries a very poor prognosis, with a median survival of ~15 months post diagnosis, and a 5-year survival rate of 5% (Ostrom et al., 2014, 2020).

Treatment options are typically limited to surgical removal of as much of the tumour mass as is safe, followed with radiotherapy, and concomitant chemotherapy using alkylating agents such as temozolomide (TMZ) (Stupp et al., 2005, 2017; Weller et al., 2017). However, TMZ, the standard-of-care, provides only marginal benefit, and is effective in less than half of GBM patients (Kamson and Grossman, 2021). Crucially, patients also acquire resistance to TMZ over time and/or in recurrent incidences of GBM (Hanif et al., 2017). Recently, an alternative strategy inhibiting tumour neoangiogenesis using bevacizumab, a humanised monoclonal antibody raised against vascular endothelial growth factor A (VEGF-A), showed no significant improvement on patient survival (Chinot et al., 2014; Gilbert et al., 2014; Kim et al., 2018), indicating the need for improved therapeutics.

The heterogenous, or ‘multiforme’, nature of GBM contributes to therapeutic failure. Whilst the neoplasms associated with the disease typically share characteristics of high invasiveness, high vascularisation, and feature hypoxic/necrotic cores (Perry and Wesseling, 2016), they exhibit substantial macroscopic, microscopic, genetic, and epigenetic discrepancies, at both the inter-and intra-tumour level (Holland, 2000).

**Figure 1:**
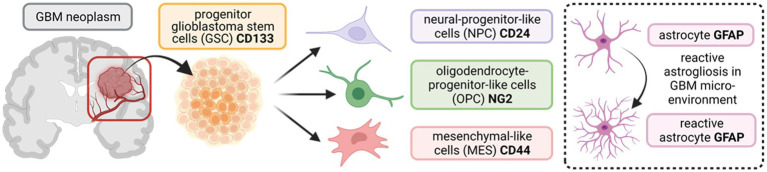
Schematic outlining the tri-lineage hierarchy of stem cell differentiation typically observed in glioblastoma (GBM) neoplasms. Three committed cell lineages, neural-progenitor-like cells (NPCs), oligodendrocyte-progenitor-like cells (OPCs), and mesenchymal-like cells (MES), originate from a common progenitor stem cell (GSC). These cells states display remarkable plasticity and are characterised by variable potential for proliferation. In addition, astrocytes in the brain parenchyma undergo reactive astrogliosis in response to insults (such as aberrant vascularisation and hypoxia in GBM), becoming reactive astrocytes (enclosed insert). Protein markers that are commonly used as targets to identify the diverse stem cell populations in immunocyto−/histo-chemical approaches are shown in bold. CD133/24/44 = Cluster of Differentiation 133//24/44; NG2 = neural/glial antigen 2; GFAP = glial fibrillary acidic protein. (Created with BioRender.com).

Diverse subsets of progenitor glioblastoma stem cells (GSCs), present in and around the tumour microenvironment, comprise some of the key regulators of GBM heterogeneity, driving tumour initiation, proliferation, invasiveness, renewal, and resistance to radio/chemotherapy (Bao et al., 2006; Chen et al., 2012; Lathia et al., 2012; Llaguno et al., 2015; Kim et al., 2021; Zheng et al., 2021). Recently, studies have used single-cell RNA sequencing, lineage tracing, and bulk genetic profiling to characterise the GSC subpopulations driving tumour heterogeneity, and a consensus is emerging that incorporates four distinct subtypes: (1) neural-progenitor-like cells (NPCs); (2) oligodendrocyte-progenitor-like cells (OPCs); (3) mesenchymal-like cells (MES); and (4) astrocytic cells, particularly reactive astrocytes that respond to insults in the tumour microenvironment (Patel et al., 2014; Neftel et al., 2019; Couturier et al., 2020; Zheng et al., 2023). A schematic of the various GSC subpopulations and antibody markers used to identify them are summarised in Figure 1. We hypothesise that identifying drug targets common across all four GSC subtypes may offer novel and effective approaches to improve therapeutic outcome in a greater number of patients.

The apelin receptor is a class A G protein-coupled receptor (GPCR), first identified based on its sequence homology with the angiotensin II type 1 receptor (O'Dowd et al., 1993), that binds two endogenous peptide ligands, apelin and elabela/Toddler (ELA) (Tatemoto et al., 1998; Chng et al., 2013; Pauli et al., 2014). Whilst only ELA acts as a key driver of proper heart formation during embryogenesis, both ligands are present in the adult cardiovascular system, where they induce potent vasodilatation and positive cardiac inotropic responses through action on the receptor present in endothelia and cardiomyocytes, respectively, (Szokodi et al., 2002; Kleinz and Davenport, 2004; Kleinz et al., 2005; Japp et al., 2008, 2010; Maguire et al., 2009; Pauli et al., 2014; Yang et al., 2017).

The apelin receptor has also garnered attention as a complex target in various cancers (Read et al., 2019; Masoumi et al., 2020), including GBM (Ivanov et al., 2022). ELA has been shown to be elevated in ovarian clear cell carcinoma (Yi et al., 2017), and, importantly, in glioma and GBM patients, where high ELA expression is directly associated with lower patient survival (Ganguly et al., 2019). Apelin was identified as a central regulator of endothelium-dependent tumour initiation and maintenance in GBM cells with stem-like properties, and the apelin receptor antagonist, MM54, was used to successfully reduce tumour expansion and lengthen survival in a GBM xenograft mouse model (Harford-Wright et al., 2017; Harford-Wright and Gavard, 2018). As further proof-of-principle, MM54 attenuates the pro-tumourigenic effects of apelin in a mouse melanoma lung metastasis model (Berta et al., 2021), suggesting that apelin receptor blocking therapy is not necessarily limited to brain cancers.

Apelin signalling has also been shown to initiate the formation of new blood vessels by driving vascular endothelial cells toward a pro-angiogenic state, in both physiological and cancerous states (Kälin et al., 2007; Yang et al., 2016; Helker et al., 2020; Berta et al., 2021). Crucially, both apelin peptide and the receptor are upregulated in GBM patient samples, and, in an orthotopic mouse model of GBM, apelin was required for tumour vascularisation, where apelin depletion significantly reduced tumour size and increased survival of tumour-bearing mice (Frisch et al., 2020). A consensus is emerging that pharmacological inhibition of the apelin receptor might offer beneficial effects on survival, either through direct actions on stem cell populations or indirectly through anti-angiogenesis or both, in various forms of cancer, including GBM (Mastrella et al., 2019; Read et al., 2019).

The expression of the apelin receptor and its endogenous ligands has not been mapped in the diverse stem cell populations in GBM that drive tumour initiation, renewal, and resistance to current therapies. Here, we used semi-quantitative fluorescence microscopy to test our hypothesis that the three key components of the apelin signalling axis (the receptor, apelin, and ELA) are expressed in progenitor GPCs, and the subsequent MES, NPC, and OPC lineages in surgical samples of primary GBM tissue from patients. Additionally, we assessed expression in microvascular proliferations in the tumour environment. Our data confirm the presence of the apelin receptor and its cognate ligands and support the potential of the apelin receptor as a drug target that is shared across the critical cell types that drive and maintain GBM.

## 2 Materials and methods

### 2.1 Materials and human tissue

All chemicals and reagents used in this study were purchased from Merck (formerly Sigma Aldrich), unless indicated otherwise. Primary and secondary antibodies used in immunohistochemistry experiments in this study are outlined in Table 1. Anonymised surgical samples of human tissue were obtained at the time of surgery, from three GBM specimens (Grade IV) from the brain and two histologically normal brain cerebral cortex (HBC) specimens. Surgical samples were collected with ethical approval and informed consent (GREF G11970; REC 5/Q0104/142) by the Human Research Tissue Bank and Cambridge Brain Bank, both supported by the NIHR Cambridge Biomedical Research Centre.

**Table 1:** Summary of the reagents and dilutions used in immunohistochemical experiments in this study.

Antibody (target)	Dilution	Species	Source	Cat. no.	Cites	CiteAb
apelin receptor	1:100	rabbit (poly)	abcam	ab84296	10	https://www.citeab.com/antibodies/711523-ab84296-anti-apj-receptor-antibody
apelin-36	1:50	rabbit (poly)	abcam	ab59469	21	https://www.citeab.com/antibodies/711409-ab59469-anti-apelin-antibody?des=e09b582419c38424
ELA-32	1:50	rabbit (poly)	Phoenix	H-007-19	3	https://www.citeab.com/antibodies/14040691-h-007-19-pglu1-ela-32-human-antibody?des=cec905b2a030cf95
CD133	1:200	mouse (mono)	abcam	ab252553	3	https://www.citeab.com/antibodies/7432801-ab252553-anti-cd133-antibody-293c3?des=39f2643b2152994a
GFAP	1:100	mouse (mono)	abcam	ab4648	126	https://www.citeab.com/antibodies/732900-ab4648-anti-gfap-antibody-2a5?des=40e015b7a7b43cc8
CD24	1:50	mouse (mono)	abcam	ab278509	1	https://www.citeab.com/antibodies/11009624-ab278509-anti-cd24-antibody-ml5?des=2ca3cb71e7dd0b08
NG2	1:100	mouse (mono)	abcam	ab50009	66	https://www.citeab.com/antibodies/769464-ab50009-anti-ng2-antibody-132-38?des=509adb83b12b9264
CD44	1:200	mouse (mono)	abcam	ab6124	87	https://www.citeab.com/antibodies/720739-ab6124-anti-cd44-antibody-f10-44-2?des=fcaa8c2c62162b7b
Ki-67	1:20	mouse (mono)	abcam	ab245113	29	https://www.citeab.com/antibodies/11010244-ab245113-anti-ki67-antibody-37c7-12?des=292322eb3ee1a248
vimentin	1:20	mouse (mono)	abcam	ab8978	659	https://www.citeab.com/antibodies/759383-ab8978-anti-vimentin-antibody-rv202-cytoskeleton?des=d55502ac6c9eeaca
von Willebrand Factor	1:50	mouse (mono)	Dako	M0616	170	https://www.citeab.com/antibodies/2414842-m0616-von-willebrand-factor-concentrate
anti-rabbit IgG AF488	1:200	donkey	abcam	ab150061	111	https://www.citeab.com/antibodies/2359550-ab150061-donkey-anti-rabbit-igg-h-l-alexa-fluor-48?des=e685c77aadc34a27
anti-mouse IgG AF555	1:200	donkey	abcam	ab150110	44	https://www.citeab.com/antibodies/2359605-ab150110-donkey-anti-mouse-igg-h-l-alexa-fluor-555?des=14ca7787136f079e
Hoechst 33342	1:1000	n/a	Invitrogen	H3570		

The species that antibodies were raised in, supplier (Source) and catalogue numbers (Cat. No.) are provided. The table also shows the number of publications the antibodies are cited in (Cites) and links to the CiteAb page that provides a summary of previous applications/publications the antibodies have been used in.

### 2.2 Immunohistochemistry

Surgical samples of human GBM Grade IV and histologically normal brain cortex (HBC) tissue were cut into 10 μm thick sections from the frozen tissue using a cryostat and thaw mounted onto microscopy slides before storage at-70°C. On the day of experimentation, slides were incubated at room temperature in a humid environment and tissue sections were ringed using a hydrophobic marker. Sections were hydrated and washed 3x with phosphate buffered saline (PBS) before fixation for ~3 min via coverage with 4% formaldehyde. Fixed sections were washed 3x with PBS and non-specific staining was blocked with PBS containing 10% donkey serum and 0.1% Tween-20 for 2 h. Blocking solution was tipped off and sections were subsequently incubated with primary antibodies for ~24 h at 4°C, prepared in PBS containing 1% donkey serum, 0.1% Tween-20, and 3.3 mg/mL bovine serum albumin (BSA). The apelin receptor, apelin, and ELA antibodies used in this study have been previously validated (Yang et al., 2017; Nyimanu et al., 2022). Controls comprised adjacent tissue sections treated with buffer in which primary antisera were omitted. Sections were washed 3x with PBS before incubation with secondary antibodies for 1 h, prepared in the same buffer as the primary antibodies. Slides were washed a further 3x with PBS before treatment with Hoechst 33342 (H3570; Invitrogen) nuclear stain for 15 min, prepared in PBS. After a final 3x washes with PBS, slides were blotted dry with lint-free tissue, mounted with ProLong Gold Antifade Mountant, covered with a cover slip, and left at room temperature in the dark to set (≥ 48 h).

### 2.3 H&E staining

Several adjacent sections of human GBM and HBC brain tissue were stained with haematoxylin and eosin (H&E), to provide greater morphological and histopathological detail. Slides were air dried for 20 min at room temperature before incubation with haematoxylin solution (Sigma; Haematoxylin Solution, Harris Modified; HHS128) for 5 min. Slides were washed in running tap water until water ran clear, incubated in Scott’s Tap Water (NaHCO₃, 3.5 g/L; MgSO₄, 20 g/L in distilled water) for 1 min and then washed in running tap water a second time before incubating with eosin solution (Sigma; Eosin Y Solution, Aqueous; HT110280) for 10 min. A final wash was performed in running tap water until water ran clear. Slides were sequentially dehydrated (2 min at each step) through an alcohol series (30, 70, and 100% ethanol), cleared with xylene for 1 h before mounting with DePeX mounting medium (Serva) and being covered with a cover slip. Slides were left to cure overnight.

### 2.4 Slide scanning imaging

For imaging and analysis, a protocol similar to that described in Williams et al., 2023 was used. Briefly, automated, high-resolution fluorescent or brightfield images (16-bit, 0.325 × 0.325 μm scaling per pixel) of immunohistochemically or H&E prepared tissue sections were acquired using an Axio Scan.Z1 slide scanner (ZEISS) with a Plan-Apochromat 20x/NA0.8 M27 objective lens, connected to a Hamamatsu Orca Flash camera. For the imaging procedure, an initial brightfield scan was performed to visualise tissue sections on the microscopy slides, and a spline contour tool was subsequently used to outline imaging regions. A profile with three fluorescent channels was used; a first channel (blue), with an LED-Module 385 nm light source set at 10% intensity and 10 ms exposure time at a depth of focus of 1.45 μm for Hoechst 33342 nuclear marker (405 nm wavelength); a second channel (green), with an LED-Module 475 nm light source set at 30% intensity and 30 ms exposure time at a depth of focus of 1.64 μm for 488 nm wavelengths; and a third channel (yellow/orange), an LED-Module 567 nm light source set at 70% intensity and 30 ms exposure time at a depth of focus of 1.88 μm for 555 nm wavelengths. Focal depths were determined automatically by the slide scanner’s in-built Z stacking autofocusing tool. Positively stained immunofluorescent samples and respective controls were all imaged using identical settings. Controls therefore provide background levels of fluorescence, encompassing autofluorescence and/or potential non-specific signal from secondary antisera, which were negligible in our samples. This was to ensure that fluorescence observed in positive samples was dependent on the presence of the primary antisera.

### 2.5 Image processing and quantification

All acquired images (.czi files) were saved and visualised using ZEN blue 3.1 software (ZEISS) and/or Orbit Image Analysis (ORBIT) software. Regions of interest (ROI) were identified using the spline contour tool, and mean fluorescence intensity (MFI) in grayscales for ROIs was provided by ZEN blue 3.1. Fluorescent signal in immunohistochemically prepared sections was deemed specific and real where it was ≥2-fold higher than that observed in corresponding ROIs in adjacent control sections where primary antisera was omitted but otherwise processed using identical conditions and at the same time to provide levels of background fluorescence. Significant differences in fluorescence intensity (grayscales) between the test samples and adjacent control sections indicated positive staining and expression of the target protein.

### 2.6 Data analysis

Quantitative data are expressed as mean ± standard deviation (SD). Graphical presentation and statistical tests were performed using GraphPad Prism version 6.07 for Windows (GraphPad Software). Statistical tests and n numbers are indicated in figure legends where applicable. A *p* value of <0.05 was used to indicate statistically significant differences.

## 3 Results

### 3.1 GBM tissue displays hallmarks of the disease

**Figure 2:**
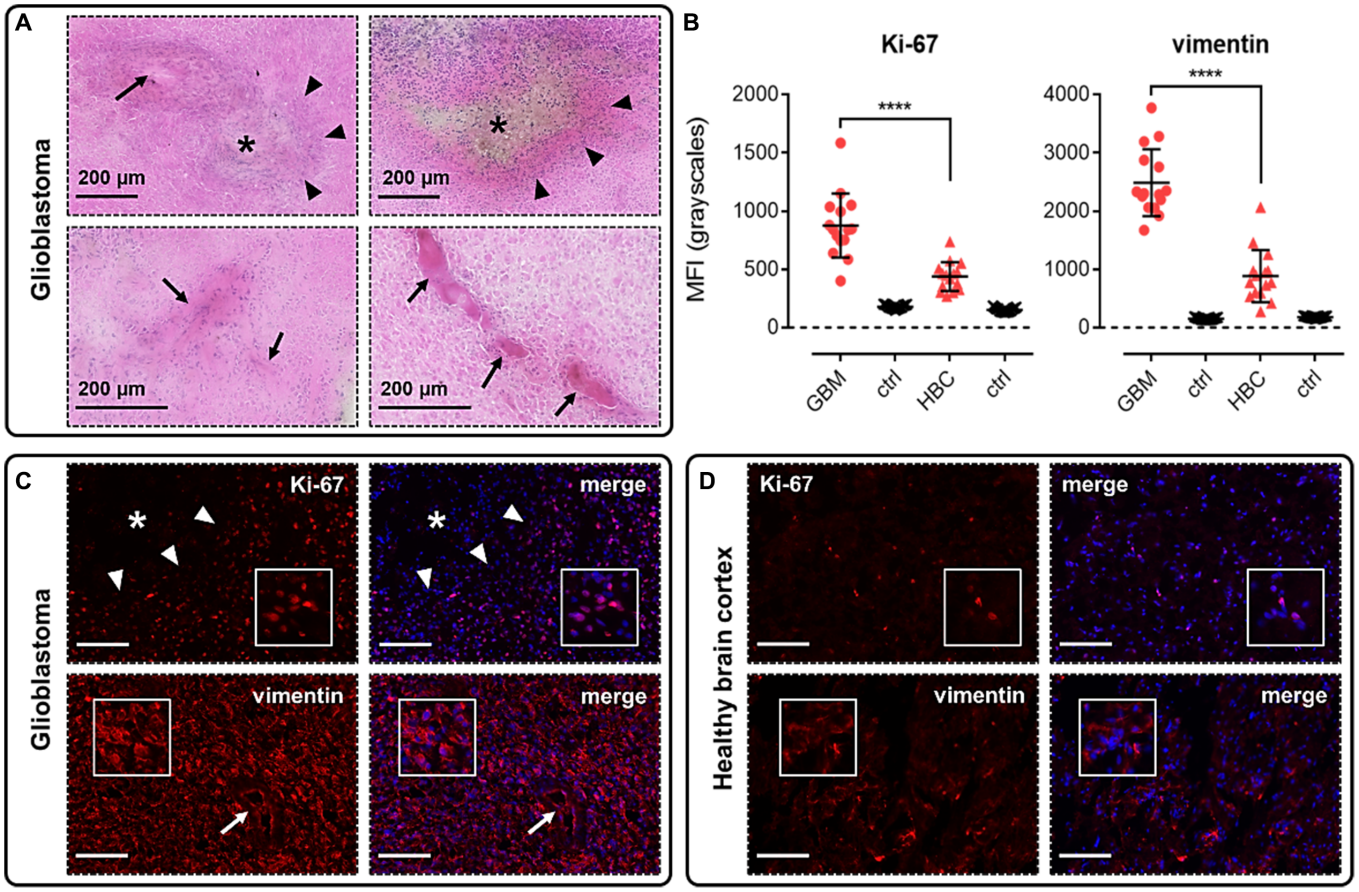
Glioblastoma (GBM) tissue used in this study displays hallmarks of the disease. **(A)** H&E staining in GBM samples allowed us to visualise hypoxic/necrotic cores (indicated by asterisks), pseudopalisades surrounding these cores (indicated by triangles), and aberrant microvascular proliferations (indicated by arrows). **(B)** Graphs showing MFI (mean fluorescence intensities, in grayscales) for staining of Ki-67 (proliferation marker) and vimentin (migration marker) in GBM versus histologically normal brain cortex (HBC) samples. Controls (ctrl) comprise adjacent tissue sections treated with primary antisera omitted. Graphical data show individual data points (*n* = 15, pooled from *n* = 3 independent donors for GBM and *n* = 2 independent donors for HBC) with mean ± SD. **** indicates *p* < 0.0001, as determined by one way ANOVA with Tukey’s correction for multiple comparisons. Representative immunofluorescent micrographs show **(C)** GBM tissue (*n* = 3 independent donors) or **(D)** HBC tissue (*n* = 2 independent donors) stained with Ki-67 or vimentin antibody, visualised in red. Merged panels show Hoechst 33342 nuclear stain, visualised in blue. Scale bars show 100 μm. Inserts show individual cells at 4x zoom.

Using H&E and immunofluorescence staining, we first confirmed that our samples of human GBM tissue displayed hallmarks of the disease, such as the formation of hypoxic/necrotic cores, associated pseudopalisades, and aberrant vascularisation (Figure 2A). GBM tissue samples were also shown to stain positively with markers for proliferation (Ki-67, Figures 2B,C) and migration (vimentin, Figures 2B,C), typical of the stem cell populations in GBM neoplasms that show strong potential for expansion and invasion. Fluorescent signal for both of these markers was significantly higher in GBM samples than in histologically normal brain cortex (HBC) samples (Figures 2B,C,D).

### 3.2 The apelin receptor is upregulated in blood vessels in GBM

**Figure 3:**
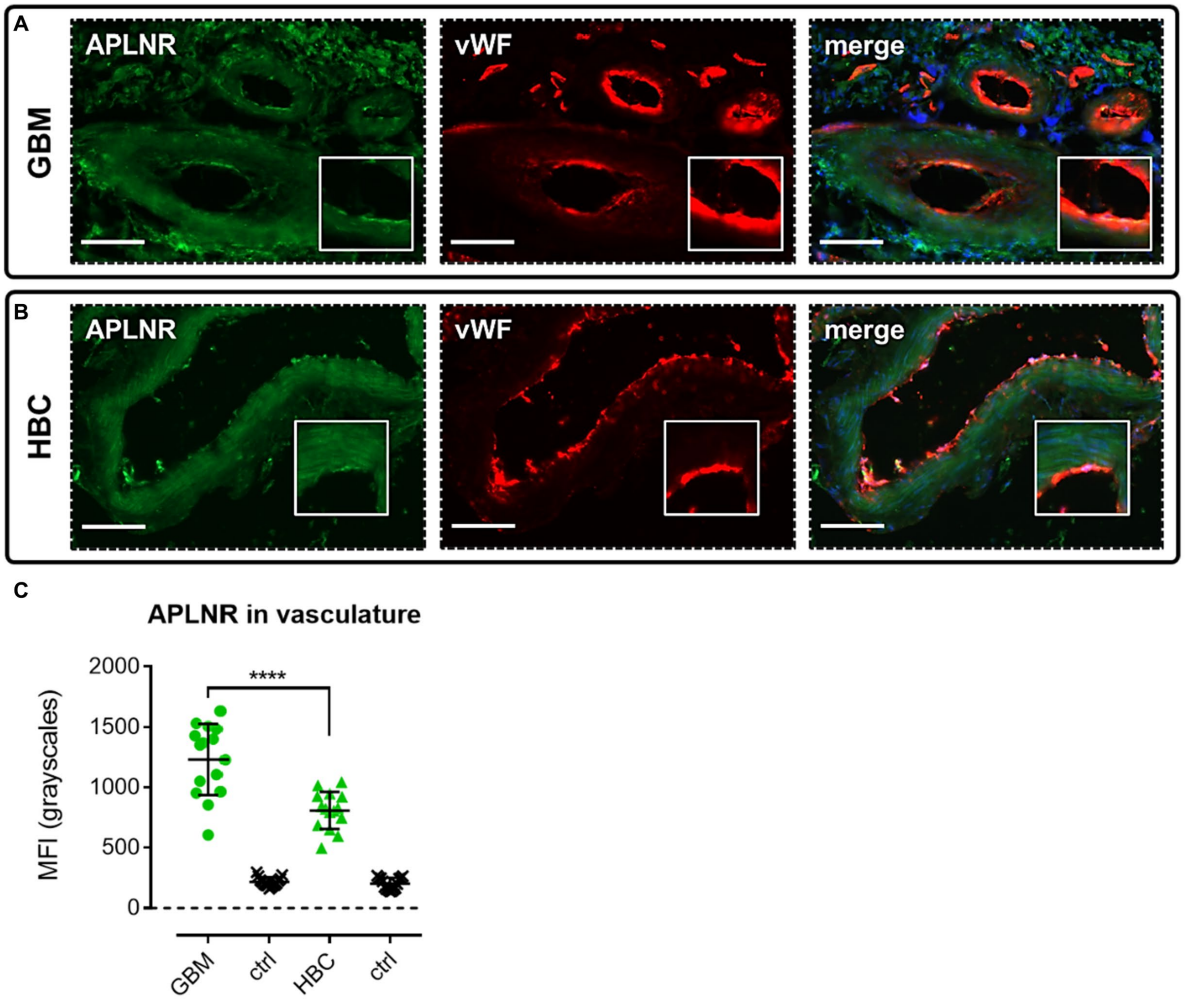
The apelin receptor (APLNR) is expressed in blood vessels in GBM. Representative immunofluorescent micrographs show blood vessels in **(A)** GBM tissue (*n* = 3 independent donors) or **(B)** histologically normal brain cortex (HBC, *n* = 2 independent donors) stained with apelin receptor antibody (visualised in green) and co-stained with von Willebrand Factor (vWF) antibody (visualised in red). Merged panels show Hoechst 33342 nuclear stain, visualised in blue. Scale bars show 100 μm. Inserts show individual cells at 4x zoom. **(C)** Graph showing MFI (mean fluorescence intensities, in grayscales) for staining of apelin receptor in GBM versus HBC samples. Controls (ctrl) comprise adjacent tissue sections treated with primary antisera omitted. Graphical data show individual data points (*n* = 15, pooled from *n* = 3 independent donors for GBM and *n* = 2 independent donors for HBC) with mean ± SD. **** indicates *p* < 0.0001, as determined by one way ANOVA with Tukey’s correction for multiple comparisons.

We performed immunohistochemical staining using apelin receptor antibody with a von Willebrand Factor antibody co-stain to assess expression of the GPCR in blood vessels in GBM and HBC samples (Figure 3). Images confirmed that the apelin receptor was expressed in the vasculature in both GBM (Figure 3A) and HBC (Figure 3B), and quantification showed that fluorescent signal for the receptor was significantly higher in GBM versus HBC (Figure 3C), suggesting that the protein is upregulated in nascent blood vessels forming in proximity to hypoxic/necrotic cores in the diseased tissue.

### 3.3 The apelin receptor and apelin peptide are expressed in progenitor GSCs

We performed immunohistochemical staining using antibodies targeting the apelin receptor and the peptides, apelin and ELA, in the presence of a panel of cell markers to assess whether this GPCR and its endogenous ligands are expressed in the diverse stem cell populations that drive glioblastoma pathogenesis and therapeutic resistance (Figure 1).

**Figure 4:**
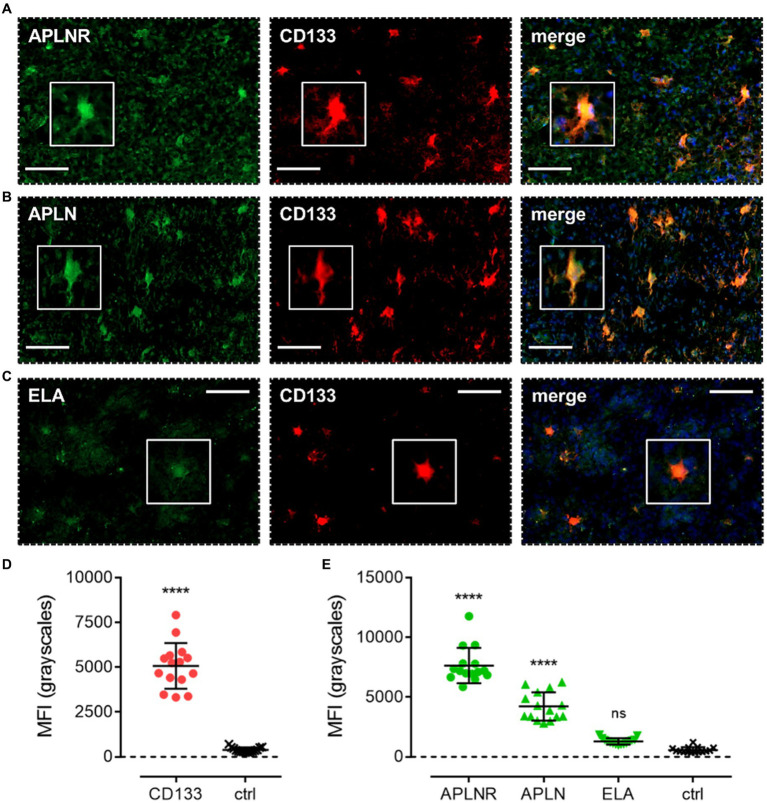
The apelin receptor and its ligands are expressed in progenitor glioblastoma stem cells (GSCs). Images show representative immunofluorescent micrographs of human glioblastoma tissue (n ≥ 3 independent donors) stained with **(A)** apelin receptor antibody (APLNR), **(B)** apelin peptide antibody (APLN), or **(C)** ELA peptide antibody (ELA), all visualised in green. Samples were co-stained with antibody raised against the GSC marker, CD133, visualised in red. Merged panels also show Hoechst 33342 nuclear stain, visualised in blue. Scale bars show 100 μm. Inserts show individual cells at 4x zoom. **(D)** MFI (mean fluorescence intensities, in grayscales) for staining of CD133-positive GSCs versus controls (ctrl, sections with primary antisera omitted). Graphical data show individual data points (*n* = 15, pooled from *n* = 3 independent donors) with mean ± SD. **** indicates *p* < 0.0001 versus ctrl, as determined by an unpaired T test. **(E)** MFI for staining of APLNR, APLN, and ELA in CD133-positive GSCs versus controls (ctrl, sections with primary antisera omitted). Graphical data show individual data points (*n* = 15, pooled from n ≥ 3 independent donors) with mean ± SD. **** indicates *p* < 0.0001, and ns indicates no significant difference, versus ctrl, as determined by one way ANOVA with Dunnett’s test for multiple comparisons.

We first characterised co-localisation of apelin receptor (Figure 4A), apelin (Figure 4B), and ELA (Figure 4C) in cells staining positive for the progenitor GSC marker, CD133. Mean fluorescence intensity (MFI, grayscales) for CD133-positive cells (5,074 ± 1,275) was significantly higher (~13-fold) than in adjacent control sections where primary antisera was omitted (392 ± 138), providing confidence in the ability of the marker to isolate the GSC population (Figure 4D). In CD133-positive GSCs, MFI for the apelin receptor (7,648 ± 1,477) and apelin peptide (4,218 ± 1,190) was significantly higher than in adjacent control sections (Figure 4E). Whilst there was a trend for higher ELA expression in CD133-positive GSCs, this was not significantly different from corresponding control sections (Figure 4E).

### 3.4 The apelin receptor, apelin, and ELA are expressed in NPCs

**Figure 5:**
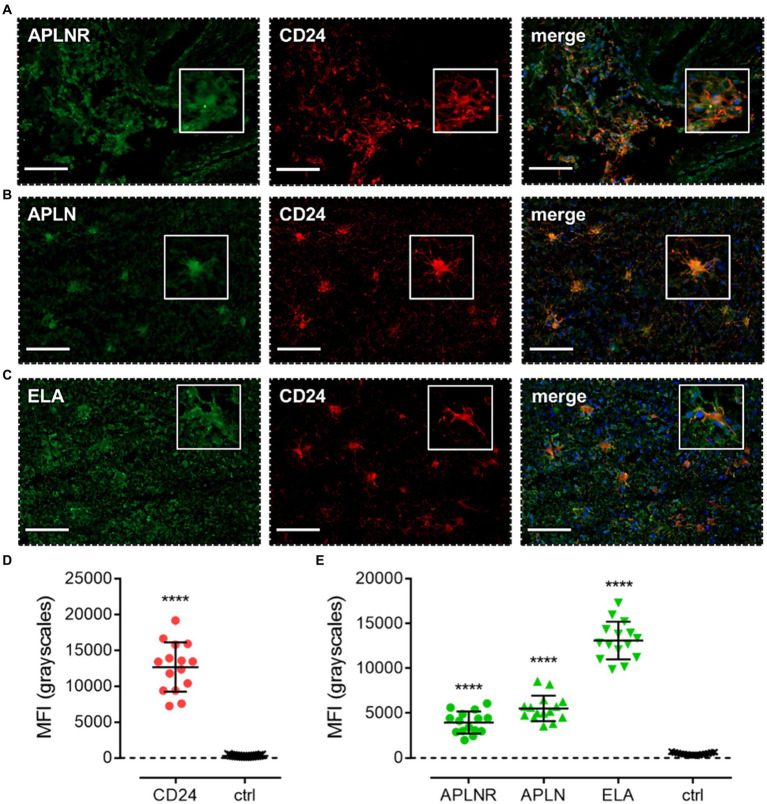
The apelin receptor and its ligands are expressed in neural-progenitor-like cells (NPCs). Images show representative immunofluorescent micrographs of human glioblastoma tissue (n ≥ 3 independent donors) stained with **(A)** apelin receptor antibody (APLNR), **(B)** apelin peptide antibody (APLN), or **(C)** ELA peptide antibody (ELA), all visualised in green. Samples were co-stained with antibody raised against the NPC marker, CD24, visualised in red. Merged panels also show Hoechst 33342 nuclear stain, visualised in blue. Scale bars show 100 μm. Inserts show individual cells at 4x zoom. **(D)** MFI (mean fluorescence intensities, in grayscales) for staining of CD24-positive NPCs versus controls (ctrl, sections with primary antisera omitted). Graphical data show individual data points (*n* = 15, pooled from *n* = 3 independent donors) with mean ± SD.**** indicates *p* < 0.0001 versus ctrl, as determined by an unpaired T test. **(E)** MFI for staining of APLNR, APLN, and ELA in CD24-positive NPCs versus controls (ctrl, sections with primary antisera omitted). Graphical data show individual data points (*n* = 15, pooled from n ≥ 3 independent donors) with mean ± SD. **** indicates *p* < 0.0001 versus ctrl, as determined by one way ANOVA with Dunnett’s test for multiple comparisons.

After demonstrating that the receptor was present in progenitor GSCs, we next looked to assess expression of the GPCR and its ligands in the committed cell lineages that originate from the GSCs. Apelin receptor (Figure 5A), apelin (Figure 5B), and ELA (Figure 5C) staining was observed to co-localise with CD24, a marker for NPCs. Again, significantly higher (~40-fold) MFI was detected in CD24-positive cells (12,670 ± 3,430) versus adjacent control sections (306 ± 89), and the presence of dendritic processes provided further evidence that the CD24 marker was accurately and specifically isolating NPCs as the cell of interest (Figure 5D). In CD24-positive cells, MFI for the apelin receptor (3,947 ± 1,231), apelin peptide (5,522 ± 1,418), and ELA peptide (13,098 ± 2096) was significantly higher than in adjacent control sections where primary antisera was omitted (464 ± 131) (Figure 5E).

### 3.5 The apelin receptor, apelin, and ELA are expressed in OPCs

**Figure 6:**
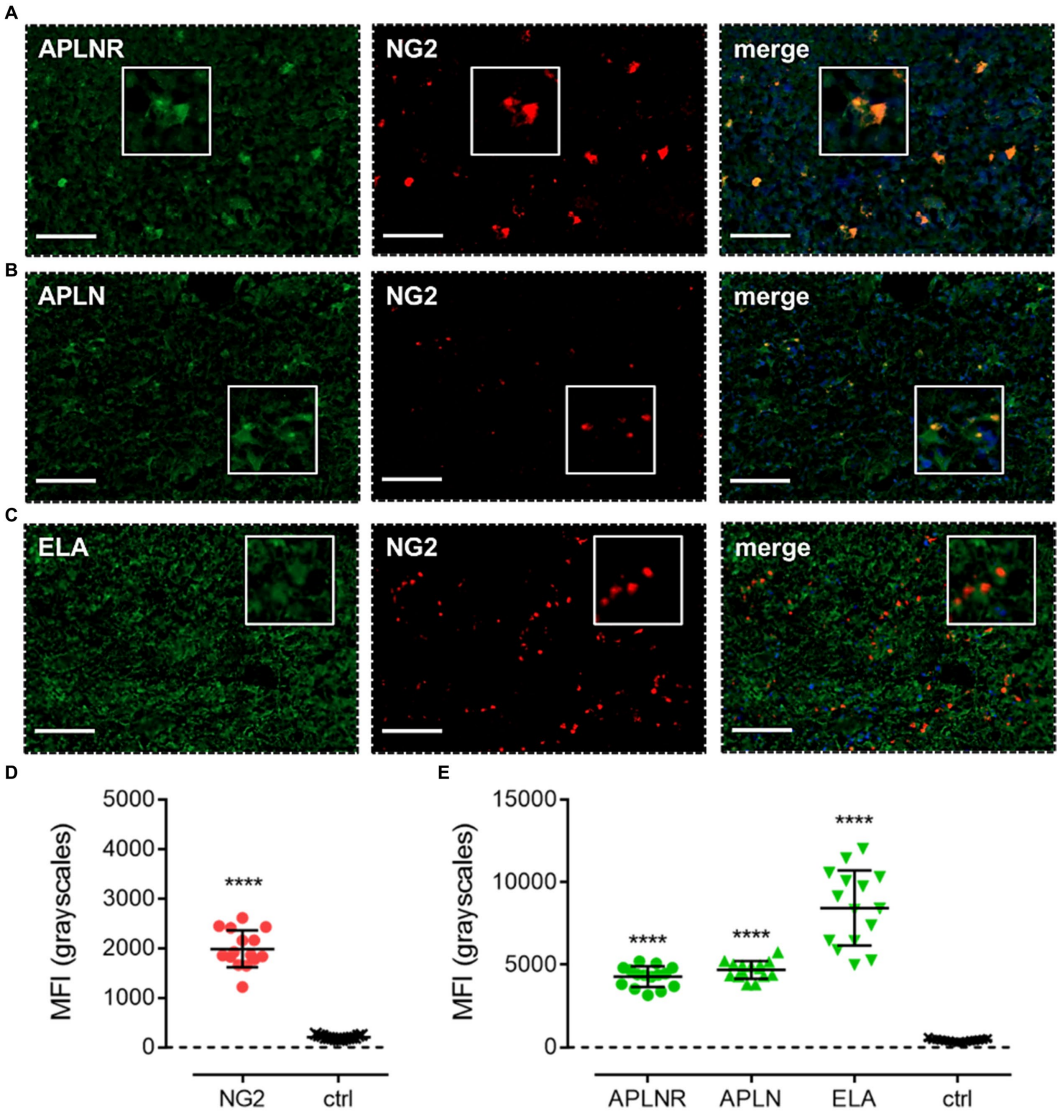
The apelin receptor and its ligands are expressed in oligodendrocyte-progenitor-like cells (OPCs). Images show representative immunofluorescent micrographs of human glioblastoma tissue (n ≥ 3 independent donors) stained with **(A)** apelin receptor antibody (APLNR), **(B)** apelin peptide antibody (APLN), or **(C)** ELA peptide antibody (ELA), all visualised in green. Samples were co-stained with antibody raised against the OPC marker, NG2, visualised in red. Merged panels also show Hoechst 33342 nuclear stain, visualised in blue. Scale bars show 100 μm. Inserts show individual cells at 4x zoom. **(D)** MFI (mean fluorescence intensities, in grayscales) for staining of NG2-positive OPCs versus controls (ctrl, sections with primary antisera omitted). Graphical data show individual data points (*n* = 15, pooled from *n* = 3 independent donors) with mean ± SD. **** indicates *p* < 0.0001 versus ctrl, as determined by an unpaired T test. **(E)** MFI for staining of APLNR, APLN, and ELA in NG2-positive OPCs versus controls (ctrl, sections with primary antisera omitted). Graphical data show individual data points (*n* = 15, pooled from n ≥ 3 independent donors) with mean ± SD. **** indicates *p* < 0.0001 versus ctrl, as determined by one way ANOVA with Dunnett’s test for multiple comparisons.

Apelin receptor (Figure 6A), apelin (Figure 6B), and ELA (Figure 6C) staining was observed to co-localise with NG2, a marker for OPCs. MFI for NG2-positive cells (1989 ± 376) was significantly higher (~10-fold) than in control tissue (203 ± 38) (Figure 6D). In NG2-positive OPCs, MFI for the apelin receptor (4,275 ± 623), apelin peptide (4,689 ± 539), and ELA peptide (8,436 ± 2,271) was significantly higher than in adjacent control tissue where primary antisera was omitted (426 ± 83) (Figure 6E). Note that, whilst the NG2 marker did not visually co-localise one-to-one with ELA in merged images (Figure 6E), the quantification demonstrates that cells that stained positively for NG2 also stained positively for ELA.

### 3.6 The apelin receptor, apelin, and ELA are expressed in MES

**Figure 7:**
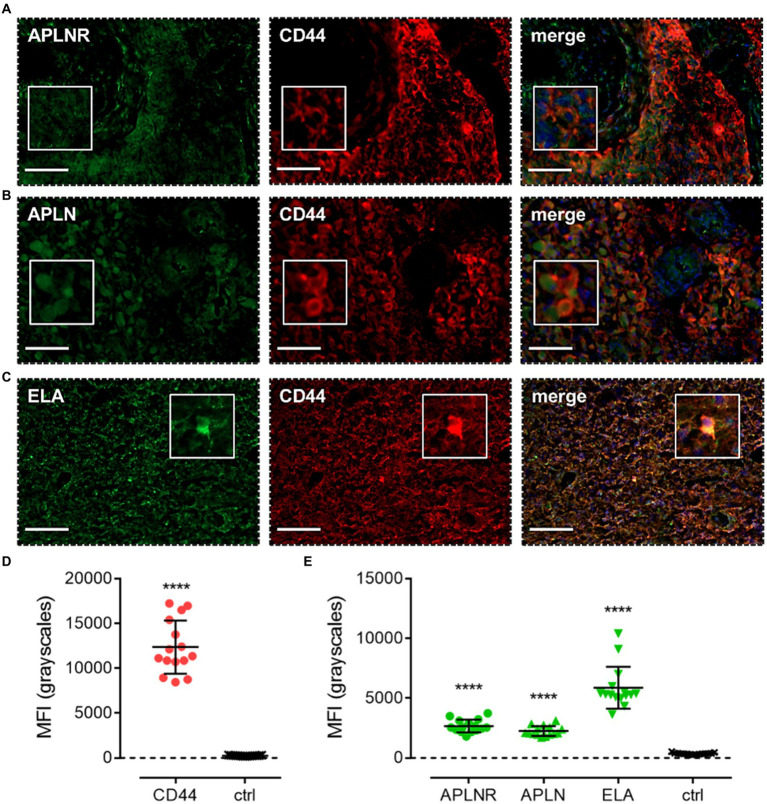
The apelin receptor and its ligands are expressed in mesenchymal-like cells (MES). Images show representative immunofluorescent micrographs of human glioblastoma tissue (n ≥ 3 independent donors) stained with **(A)** apelin receptor antibody (APLNR), **(B)** apelin peptide antibody (APLN), or **(C)** ELA peptide antibody (ELA), all visualised in green. Samples were co-stained with antibody raised against the MES marker, CD44, visualised in red. Merged panels also show Hoechst 33342 nuclear stain, visualised in blue. Scale bars show 100 μm. Inserts show individual cells at 4x zoom. **(D)** MFI (mean fluorescence intensities, in grayscales) for staining of CD44-positive MES versus controls (ctrl, sections with primary antisera omitted). Graphical data show individual data points (*n* = 15, pooled from *n* = 3 independent donors) with mean ± SD. **** indicates *p* < 0.0001 versus ctrl, as determined by an unpaired T test. **(E)** MFI for staining of APLNR, APLN, and ELA in CD44-positive MES versus controls (ctrl, sections with primary antisera omitted). Graphical data show individual data points (*n* = 15, pooled from n ≥ 3 independent donors) with mean ± SD. **** indicates *p* < 0.0001 versus ctrl, as determined by one way ANOVA with Dunnett’s test for multiple comparisons.

Apelin receptor (Figure 7A), apelin (Figure 7B), and ELA (Figure 7C) staining was also observed to co-localise with CD44, a marker for MES cells. MFI for CD44-positive cells (12,368 ± 2,965) was significantly higher (~50-fold) than in adjacent control sections where primary antisera was omitted (251 ± 45) (Figure 7D). In CD44-positive MES cells, MFI for the apelin receptor (2,672 ± 530), apelin peptide (2,261 ± 410), and ELA peptide (5,871 ± 1751) was significantly higher than in the control sections (363 ± 68) (Figure 7E).

### 3.7 The apelin receptor, apelin, and ELA are expressed in astrocytes

**Figure 8:**
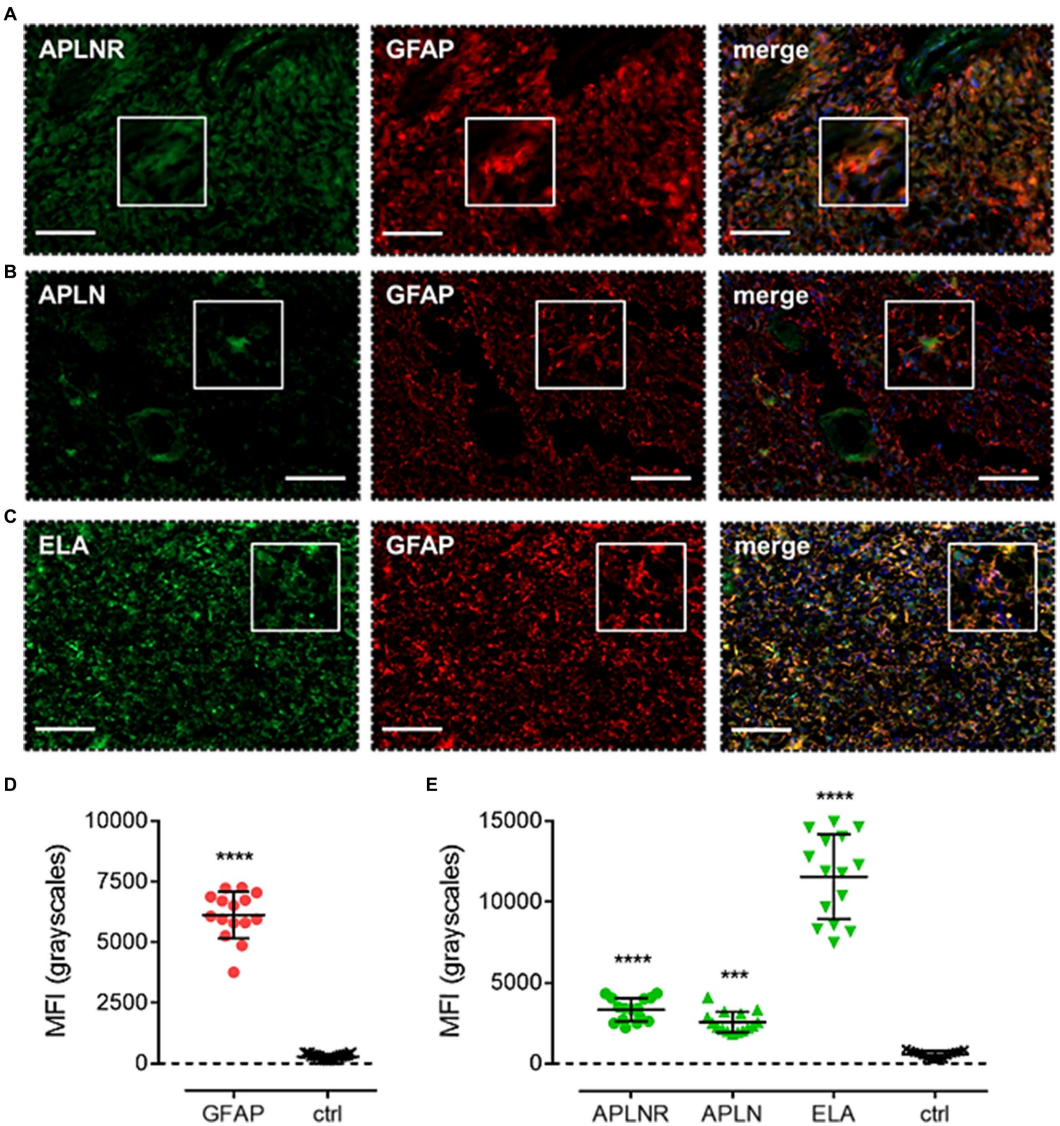
The apelin receptor and its ligands are expressed in reactive astrocytes. Images show representative immunofluorescent micrographs of human glioblastoma tissue (n ≥ 3 independent donors) stained with **(A)** apelin receptor antibody (APLNR), **(B)** apelin peptide antibody (APLN), or **(C)** ELA peptide antibody (ELA), all visualised in green. Samples were co-stained with antibody raised against the astrocytic marker, GFAP, visualised in red. Merged panels also show Hoechst 33342 nuclear stain, visualised in blue. Scale bars show 100 μm. Inserts show individual cells at 4x zoom. **(D)** MFI (mean fluorescence intensities, in grayscales) for staining of GFAP-positive astrocytes versus controls (ctrl, sections with primary antisera omitted). Graphical data show individual data points (*n* = 15, pooled from *n* = 3 independent donors) with mean ± SD. **** indicates *p* < 0.0001 versus ctrl, as determined by an unpaired T test. **(E)** MFI for staining of APLNR, APLN, and ELA in GFAP-positive astrocytes versus controls (ctrl, sections with primary antisera omitted). Graphical data show individual data points (*n* = 15, pooled from *n* = 3 independent donors) with mean ± SD. **** indicates *p* < 0.0001, *** indicates *p* < 0.001 versus ctrl, as determined by one way ANOVA with Dunnett’s test for multiple comparisons.

Finally, apelin receptor (Figure 8A), apelin (Figure 8B), and ELA (Figure 8C) staining was assessed in astrocytes, particularly those that typically undergo reactive astrogliosis when exposed to insults originating from hypoxic/necrotic cores and aberrant blood vessels that comprise some of the hallmarks of GBM pathogenesis. We used GFAP to identify astrocytes and selected regions in proximity to qualitatively identified cores and/or microvascular proliferations. MFI for GFAP-positive cells (6,123 ± 960) was significantly higher (~21-fold) than in adjacent control sections where primary antisera was omitted (289 ± 103) (Figure 8D). In GFAP-positive astrocytes, MFI for the apelin receptor (3,335 ± 721), apelin peptide (2,561 ± 638), and ELA peptide (11,559 ± 2,622) was significantly higher than in control sections (569 ± 223) (Figure 8E). Our findings for the expression of apelin receptor, apelin peptide, and ELA peptide in the key, diverse GBM stem cell populations are summarised in Table 2.

**Table 2:** Summary of the diverse stem cell and astrocytic populations that drive GBM pathogenesis and therapeutic resistance, associated cell markers, and relative co-expression of the apelin receptor or its two endogenous peptide ligands, apelin and ELA.

GBM cell type	Stem cell marker	Apelin receptor	Apelin	ELA
GSCs 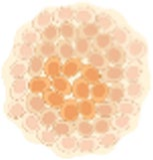	CD133	++	+	ns
NPCs 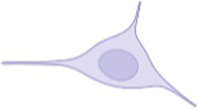	CD24	+	++	+++
OPCs 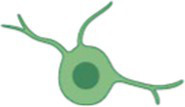	NG2	+	+	++
MES 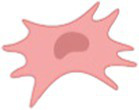	CD44	+	+	++
Astrocytes 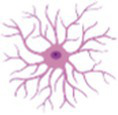	GFAP	+	+	+++

MFI ≥ 2000 grayscales (+), ≥ 5,000 grayscales (++), ≥ 10,000 grayscales (+++), ns indicates no significant difference versus MFI in adjacent control sections. (Cell images created with BioRender.com).

## 4 Discussion

GBM is characterised by pathological hallmarks, such as hypoxic/necrotic cores and aberrant microvascular proliferations (Perry and Wesseling, 2016), which we were consistently observed in the human tissue samples used in this study (Figure 2A). We were also able to show that these GBM samples typically expressed Ki-67 and vimentin (markers of proliferation and potential migration, respectively) at higher levels than in HBC (Figures 2B,C,D), as might be expected in cancerous tissue.

The apelin receptor has emerged as a potential therapeutic target in various cancers, including GBM (Read et al., 2019; Masoumi et al., 2020; Ivanov et al., 2022). Apelin signalling is known to drive formation of new blood vessels in both physiology and disease (Kälin et al., 2007; Yang et al., 2016; Helker et al., 2020; Berta et al., 2021), and it has recently been shown that apelin receptor knockdown or receptor inhibition with a peptide antagonist reduces vascularisation and angiogenesis in GBM (Mastrella et al., 2019).

In this study we visualised and measured intense immunofluorescence with antisera to apelin receptor and its two endogenous ligands in GBM tissue. These were chosen as they have been extensively validated by other researchers as indicated in Table 1. We used adjacent sections with primary antisera omitted to measure autofluorescence, although a limitation is that the small samples available precluded using additional isotype controls.

We have shown that blood vessels in GBM tissue express the apelin receptor (Figure 3A), expanding on previous findings showing expression of apelin peptide in GBM tumour blood vessels (Harford-Wright et al., 2017). In our study, vascular expression of the apelin receptor was significantly higher in GBM versus HBC (Figures 3B,C), identifying an upregulation of the receptor in the vasculature in the cancerous state. Overall, the data confirm that the apelin receptor is present as a potential vascular anti-angiogenic drug target in GBM.

The pathological role of the apelin receptor and its endogenous peptide ligands, apelin and ELA, in cancers such as GBM may well extend beyond angiogenesis. In this study, we used a panel of cell markers to assess expression of the apelin signalling axis in the four distinct GSC lineages – NPCs, OPCs, MES, and astrocytes – that have emerged as the key drivers of GBM proliferation, maintenance, invasion, and therapeutic resistance (Patel et al., 2014; Neftel et al., 2019; Couturier et al., 2020; Zheng et al., 2023).

First, co-staining with CD133 showed that both the apelin receptor and apelin peptide were expressed in GSCs (Figures 4A,B,E), the cells from which the three lineages originate. Interestingly, however, we did not observe expression of ELA peptide in these cells (Figures 4C,E). Apelin peptide expression has been demonstrated in GSCs previously and apelin receptor inhibition has been shown to blunt GSC invasion (Mastrella et al., 2019). Our work is important in providing evidence that this blunting effect on pro-invasiveness might be mediated directly through apelin receptor expression in GSCs.

Next, co-staining with CD24, NG2, CD44, and GFAP isolated NPCs, OPCs, MES, and astrocytic cells, respectively. In each of these cell subtypes, we observed significant levels of apelin receptor, apelin peptide, and ELA peptide (Figures 5, 6, 7, 8; Table 2). It is interesting that ELA was present in the three lineages and astrocytes but not the originating GSCs, and further work will need to be done to characterise whether ELA plays a role as a potential phenotypic switch in stem cell differentiation in the brain. Nevertheless, our data demonstrate that the apelin receptor and its ligands are commonly present across the key GBM cell subtypes.

In terms of future work and perspectives, we suggest that it would be of great interest to further employ *in vivo* xenograft models (Harford-Wright et al., 2017; Harford-Wright and Gavard, 2018), or use vascularised brain organoid models (Sun et al., 2022) that incorporate the necessary stromal cells in the microenvironment, such as macrophages, to investigate the function of the apelin receptor in GBM and other gliomas. These brain tumour models could also act as ideal platforms for the screening of potential drugs targeting the apelin signalling axis.

[Pyr^1^] apelin infused systemically into volunteers (Japp et al., 2008) has been shown to be safe and well tolerated, even at the highest concentration of 135 nmol/min for 120 min (Nyimanu et al., 2019). A similar safety profile was also reported for the G-protein biassed agonist MM07 (Brame et al., 2015) and the small molecule agonist (Winkle et al., 2023) as well as for [Pyr^1^] apelin in obesity (Castan-Laurell et al., 2008) and patients with heart failure (Japp et al., 2010). The results with agonists demonstrate apelin is limited to two principal actions in humans in health and disease: modest vasodilatation within the first hour of systemic infusion and a beneficial increase in cardiac output that is maintained for up to six hours in patients with heart failure (Barnes et al., 2013).

Our therapeutic hypothesis is that the apelin receptor contributes to the development and progression of glioblastoma, and supported by results from animal models, apelin antagonist may act in combination with current standard-of-care to improve the treatment of this condition. Although the precise mechanism of action has to be determined, we have shown all cell types present in GBM that have been implicated in tumour progression, express the apelin receptor. We further hypothesise that an antagonist would be expected to be effective against all these cell types. The apelin antagonist MM54 has been shown to be effective in a mouse models of GBM (Harford-Wright et al., 2017) and a mouse melanoma lung metastasis model (Berta et al., 2021). MM54 has been tested for potential toxic effects *in vivo* using mice. MM54 was administered *in vivo* at the same concentration (2 mg/kg) that was effective in slowing the progression of glioblastoma patient-derived cells with stem-like properties. There were no adverse changes to cardiac frequency, blood pressure or glycaemia, or pathological changes in the heart, kidney, aorta, or liver. The results showed MM54 was safe and effective in reducing tumour expansion and lengthening the survival of intracranially xenografted mice (Harford-Wright et al., 2017). To date there have been no clinical trials of apelin antagonists in humans. It is not yet known if apelin contributes to vascular tone or maintenance of cardiac output but unwanted side effects, such as increase in blood pressure, if they occur, may be reduced by existing anti-hypertensive therapies.

In summary, whilst we have not examined molecular mechanisms underlying apelin receptor signalling in GBM in this study, we present a descriptive analysis of receptor and ligand expression in the vasculature, GSCs, and the four key cell types that drive GBM tumour proliferation, invasiveness, maintenance, and resistance to current therapies. The demonstration of the presence of the apelin receptor and ligands in the diverse GSC and astrocytic populations holds therapeutic promise as a common druggable target in an otherwise heterogenous microenvironment. Targeting the apelin receptor may present a new treatment option, which blunts GSC proliferation and pathological angiogenesis, to synergise with current therapies such as TMZ to improve patient outcome in GBM.

## Data availability statement

The raw data supporting the conclusions of this article will be made available by the authors, without undue reservation.

## Ethics statement

The studies were conducted in accordance with the local legislation and institutional requirements. The patients provided their written informed consent to participate in this study. The human samples used in this study were acquired from surgical samples of human tissue that were collected with ethical approval and informed consent (GREF G11970; REC 05/Q0104/142) by the Human Research Tissue Bank and Cambridge Brain Bank.

## Author contributions

TW: Conceptualization, Writing – original draft, Writing – review & editing, Formal analysis, Investigation, Methodology. PN: Conceptualization, Formal analysis, Investigation, Methodology, Writing – original draft, Writing – review & editing. RK: Formal analysis, Investigation, Methodology, Writing – original draft, Writing – review & editing. KS: Formal analysis, Investigation, Methodology, Writing – original draft, Writing – review & editing. AP: Formal analysis, Writing – original draft, Writing – review & editing. KA: Formal analysis, Writing – original draft, Writing – review & editing. JM: Formal analysis, Writing – original draft, Writing – review & editing, Conceptualization, Funding acquisition, Supervision. AD: Conceptualization, Funding acquisition, Supervision, Writing – original draft, Writing – review & editing, Resources.
